# Single-layer perceptron artificial visual system for orientation detection

**DOI:** 10.3389/fnins.2023.1229275

**Published:** 2023-08-22

**Authors:** Hiroyoshi Todo, Tianqi Chen, Jiazhen Ye, Bin Li, Yuki Todo, Zheng Tang

**Affiliations:** ^1^Wicresoft Co., Ltd, Tokyo, Japan; ^2^Division of Electrical Engineering and Computer Science, Kanazawa University, Kanazawa, Japan; ^3^Chengfang Financial Information Technology Service Corporation, Beijing, China; ^4^Department of Intelligence Information Systems, University of Toyama, Toyama, Japan

**Keywords:** perceptron, single-layer, visual system, orientation detection, computer vision

## Abstract

Orientation detection is an essential function of the visual system. In our previous works, we have proposed a new orientation detection mechanism based on local orientation-selective neurons. We assume that there are neurons solely responsible for orientation detection, with each neuron dedicated to detecting a specific local orientation. The global orientation is inferred from the local orientation information. Based on this mechanism, we propose an artificial visual system (AVS) by utilizing a single-layer of McCulloch-Pitts neurons to realize these local orientation-sensitive neurons and a layer of sum pooling to realize global orientation detection neurons. We demonstrate that such a single-layer perceptron artificial visual system (AVS) is capable of detecting global orientation by identifying the orientation with the largest number of activated orientation-selective neurons as the global orientation. To evaluate the effectiveness of this single-layer perceptron AVS, we perform computer simulations. The results show that the AVS works perfectly for global orientation detection, aligning with the majority of physiological experiments and models. Moreover, we compare the performance of the single-layer perceptron AVS with that of a traditional convolutional neural network (CNN) on orientation detection tasks. We find that the single-layer perceptron AVS outperforms CNN in various aspects, including identification accuracy, noise resistance, computational and learning cost, hardware implementation feasibility, and biological plausibility.

## 1. Introduction

A hyper-complex neural network, consisting of approximately 10^11^ neurons and over 10^15^ interconnections, facilitates the timely reception and processing of information from the eyes, ears, nose, and skin within our brain (Todo et al., 2019). Visual stimuli account for more than 80 percent of the information received when our eyes are open, and nearly 50 percent of nerve fibers are directly or indirectly associated with the retina (Medina and Hanlon, 2009; Lee et al., 2020). The visual system primarily focuses on contrast, color, and movement changes, all of which have the potential to influence human behavior (Vanston and Strother, 2017). Therefore, studying the visual system is crucial for unraveling the workings of the brain. Between 1950 and 1980, Canadian neurophysiologist David Hubel and Swedish neuroscientist Torsten Wiesel conducted meticulous and scientific investigations into the visual mechanism. Their research and experiments on cortex cells in rabbits and monkeys led to the observation of several biological phenomena: (1) visual cortex cells exhibit specific responses to rectangular light spots and slits, and (2) there are simple cortical cells in the visual cortex that respond exclusively to stimuli of particular angles within their receptive fields (Hubel and Wiesel, 1959, 1962, 1968; Hubel, 1982). These neurons possess orientation selectivity, firing preferentially in response to specific orientations while exhibiting little to no response to others. Orientation detection constitutes a fundamental function of the visual system, aiding us in recognizing our surroundings and making judgments and decisions. However, our understanding of orientation selectivity and its role in global orientation detection for objects of various sizes, shapes, and positions remains limited (Gazzaniga, 2000; Veeser and Cumming, 2017). To address this issue, we presented a novel and comprehensive mechanism elucidating global visual orientation detection in our previous paper (Li et al., 2021). Based on this mechanism, we introduced a single-layer perceptron Artificial Visual System (AVS) for global orientation detection. This AVS implements local orientation-selective neurons using a single-layer perceptron composed of McCulloch-Pitts neurons. Each neuron is responsible for detecting a specific orientation angle within a two-dimensional local receptive field. The design of weights and thresholds for the single-layer perceptron is straightforward, drawing upon our knowledge of perceptron and local orientation-detective neurons. The global orientation of an object can be inferred by identifying the orientation-selective neuron with the highest number of activations. To validate the effectiveness of the single-layer perceptron AVS in determining the global orientation of objects, we conducted computer simulations using an image dataset. The results of these simulations demonstrate that the single-layer perceptron AVS is highly effective, accurately discerning the global orientation of objects regardless of their size, shape, or position. These findings align with the majority of physiological experiments and models. Moreover, to highlight the superiority of the single-layer perceptron AVS, we compared its performance with that of a traditional Convolutional Neural Network (CNN) in global orientation detection tasks. Remarkably, the single-layer perceptron AVS outperformed the CNN in all aspects, including identification accuracy, noise resistance, computational and learning costs, hardware implementation feasibility, as well as biological soundness and reasonability.

## 2. System

### 2.1. Single-layer perceptron

**Figure 1:**
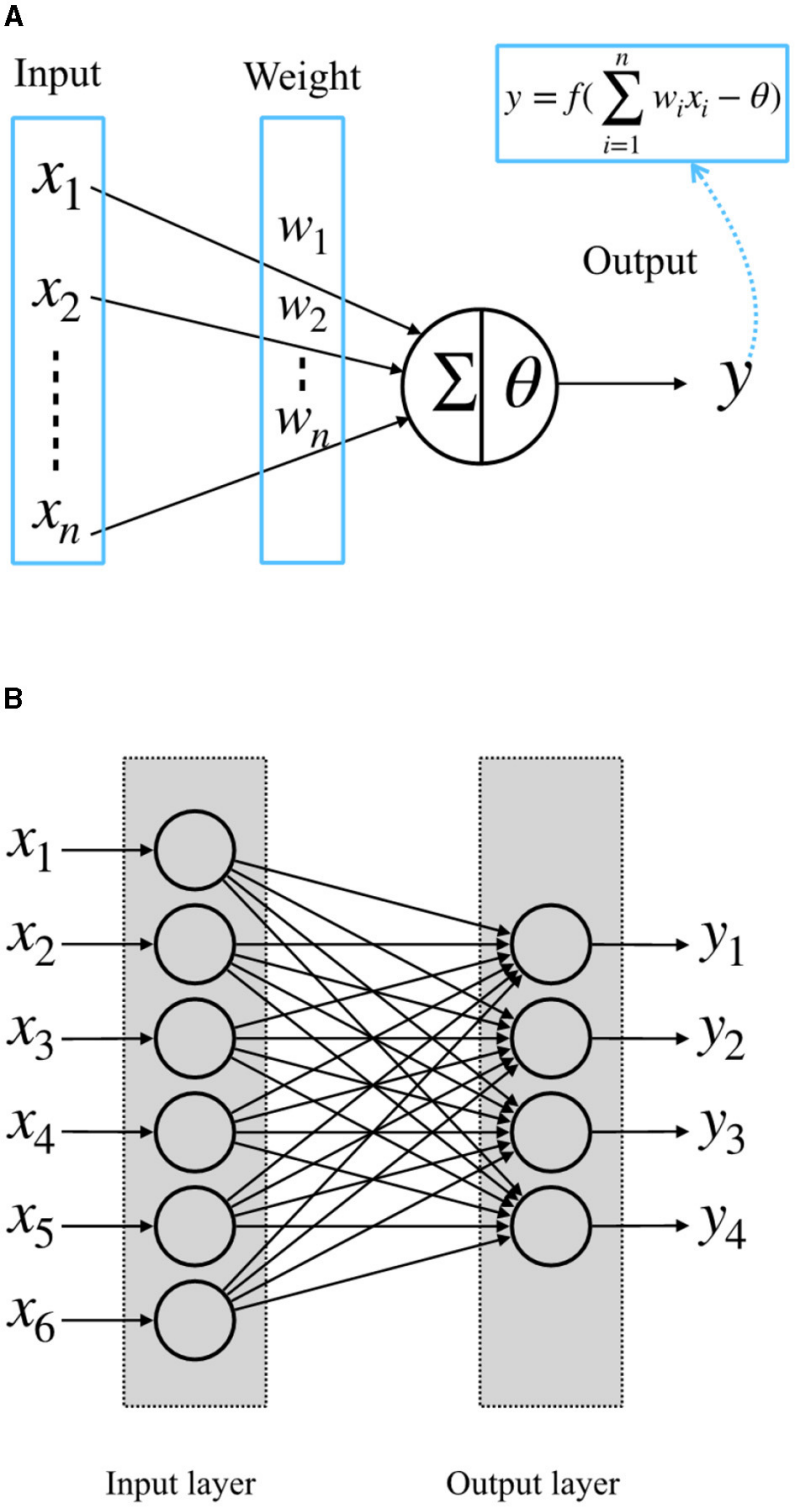
**(A)** McCulloch-Pitts neuron model; **(B)** a single-layer perceptron.

McCulloch-Pitts artificial neuron model was proposed in the 1940s (McCulloch and Pitts, 1943). It is a simple model of biological nerve cells. The structure of the McCulloch-Pitts model is shown in Figure 1A. In this model, the neuron receives input signals *x*_1_, *x*_2_,..., *x*_*n*_ from other neurons. The importance of these input signals is usually represented by the weights of the connections between neurons, *w*_1_, *w*_2_,..., *w*_*n*_. The neuron multiplies the received input values with the corresponding weights, sums them to get value ∑*w*_*i*_*x*_*i*_, and compares it with a threshold θ. When the sum exceeds the threshold, the neuron fires to output *y* = 1; otherwise, *y* = 0 (McCulloch and Pitts, 1943). When several such neurons are combined into a system, as shown in Figure 1B, we call it a perceptron, or a single-layer perceptron, which consists of a single layer of the McCulloch-Pitts neurons connected to a set of inputs from other neurons with their own weights (Rosenblatt, 1958).

### 2.2. Local orientation-selective neuron

**Figure 2:**
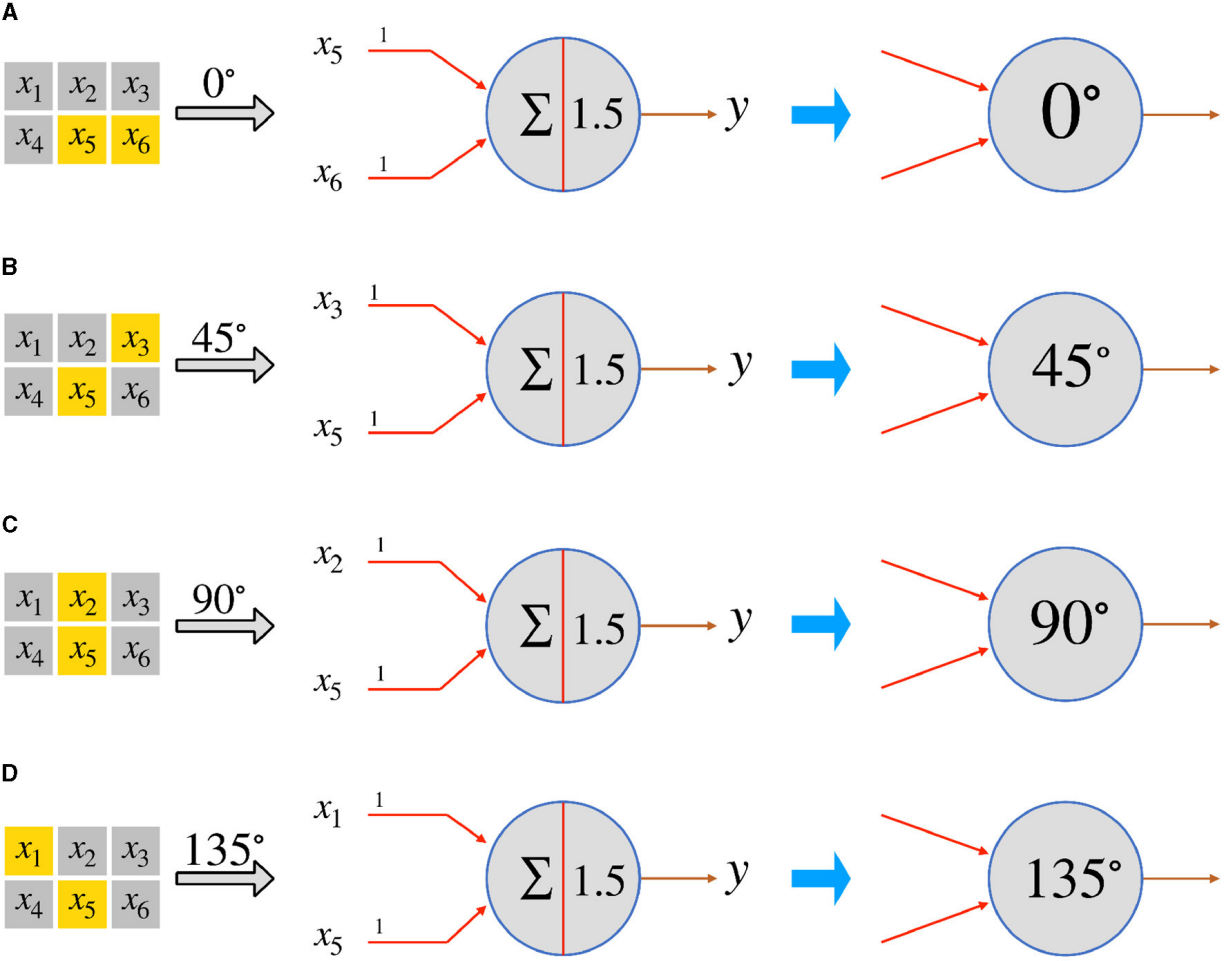
The perceptrons for the four types of orientation-selective neurons in a 3 × 2 local receptive field. **(A)** 0°-selective neuron, **(B)** 45°-selective neuron, **(C)** 90°-selective neuron, and **(D)** 135°-selective neuron.

In this subsection, we use the single-layer perceptron to realize local orientation-selective neurons. We postulate the existence of numerous simple neurons that possess orientation detection capabilities. Each neuron is solely responsible for detecting a specific orientation within a small area known as the local receptive field of the overall visual field. For the sake of simplicity, let's consider a 3 × 2 local receptive field. Within this field, there are four possible orientation angles: 0°, 45°, 90°, and 135°, that can be detected. By employing four McCulloch-Pitts neurons, we can realize four distinct types of orientation-selective neurons that can detect two-dimensional objects with orientation angles of 0°, 45°, 90°, and 135°, respectively. In the actual visual system, the primary pathway for transmitting visual information follows the sequence: photoreceptor → bipolar cell → ganglion cell → lateral geniculate nucleus (LGN) → primary visual cortex (Kandel et al., 1991). Considering a two-dimensional visual field, or the receptive field, we assume that it can be divided into M × N regions. Each region corresponds to the smallest visually distinguishable area. When light falls on a region, the corresponding photoreceptor, or a bunch of photoreceptors, converts the light signals into electrical signals, which are then transmitted to bipolar cells. To simplify the neural computation, we focus solely on the ON-response mechanism. Consequently, if a photoreceptor receives light, its corresponding ON-response bipolar cell outputs 1; otherwise, it outputs 0. For the sake of simplicity, we directly connect the photoreceptors to the orientation-selective ganglion neurons. Each type of orientation-selective ganglion neuron accepts signals from the corresponding bipolar cell or photoreceptor, based on its specific orientation selectivity. By considering *x*_5_ as the reference point, we can establish the corresponding connections for the four types of orientation-selective neurons within a local receptive field consisting of six (3 × 2) regions, as depicted in Figure 2.

**Figure 3:**
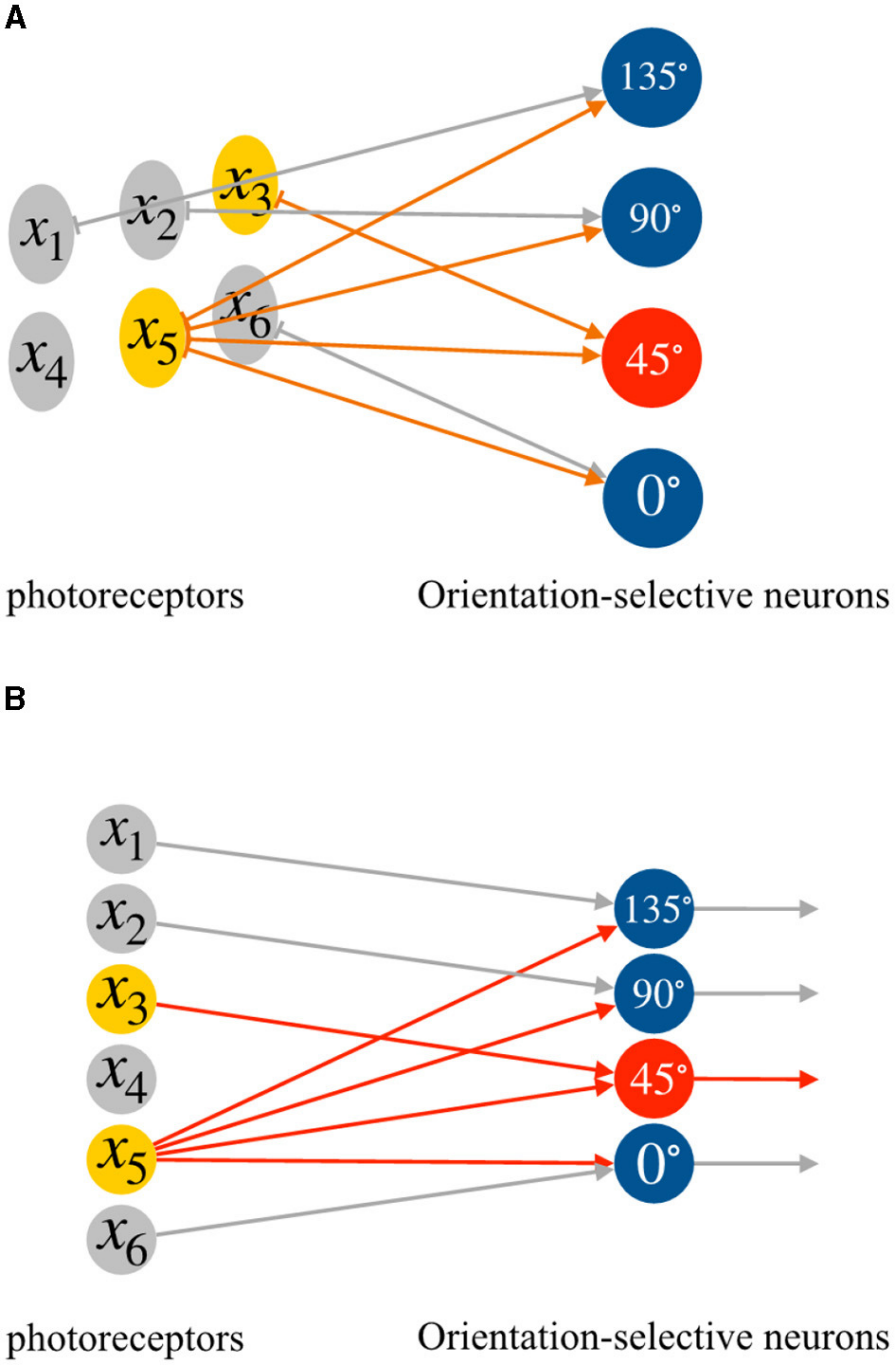
The neural connections in a local receptive field, **(A)** the connections between photoreceptors and orientation-selective neurons, **(B)** the perceptron form of the connections between photoreceptors and orientation-selective neurons.

By examining Figure 2, we can determine that the size of the local receptive field is set as 3 × 2, and the orientation-selective neurons respond to two inputs. Within the 3 × 2 local receptive field, the input signals are labeled from *x*_1_ to *x*_6_, with *x*_5_ serving as the reference point. Consequently, the 0°-selective neuron only responds to inputs *x*_5_ and *x*_6_, the 45°-selective neuron exclusively responds to *x*_3_ and *x*_5_, the 90°-selective neuron solely responds to *x*_2_ and *x*_5_, and the 135°-selective neuron responds solely to *x*_1_ and *x*_5_. As the photoreceptors output 1 when they receive light, and 0 otherwise, and the weights (from *w*_1_ to *w*_5_) are all set to 1, a neuron will only fire if both of its input signals from the photoreceptors are 1 simultaneously. Thus, we can set the threshold as 1.5 and adopt a step function as the activation function *f*:


(1) $$\begin{array}{l} y=\{\begin{array}{c} 1,(w_{i}x_{i}+w_{j}x_{j}\geq 1.5)\\ 0,(w_{i}x_{i}+w_{j}x_{j}<1.5) \end{array}\;, \end{array}$$

where *x*_*i*_ and *x*_*j*_ represent the two effective inputs, *w*_*i*_ and *w*_*j*_ are their corresponding weights. The effective input information varies for different orientation-selective neurons. As a result, we connect each corresponding region to four distinct orientation-selective neurons. Figure 3 illustrates an example of the connections between the photoreceptors and the four different orientation-selective neurons within a local receptive field. In Figure 3A, for a 3 × 2 local receptive field, only one region, the reference region, is connected to all orientation-selective neurons. The other four photoreceptors are connected to their corresponding orientation-selective neurons. Each orientation-selective neuron focuses on specific outputs from the photoreceptors and accepts the corresponding orientation information. If we represent the neural connections within a local receptive field in the form of a perceptron, the perceptron AVS can be visualized as shown in Figure 3B. It is worth noting that by considering only four orientations, the system takes advantage of the symmetrical relationships:

0° and 180°: These orientations are mirror images of each other. Any pattern or object rotated by 180° will look identical to the original pattern.

45° and 225°: Similarly, these orientations are mirror images of each other. Any pattern or object rotated by 225° will look identical to the original pattern.

90° and 270°: These orientations are perpendicular to each other. A pattern or object rotated by 90° becomes the same as the original pattern.

135° and 315°: These orientations are also perpendicular and mirror images of each other. A pattern or object rotated by 315° will look identical to the original pattern.

By considering only one orientation from each symmetrical pair, the system avoids redundancy and reduces computational complexity while still being able to capture the essential information needed for orientation detection.

### 2.3. Global orientation detection system

**Figure 4:**
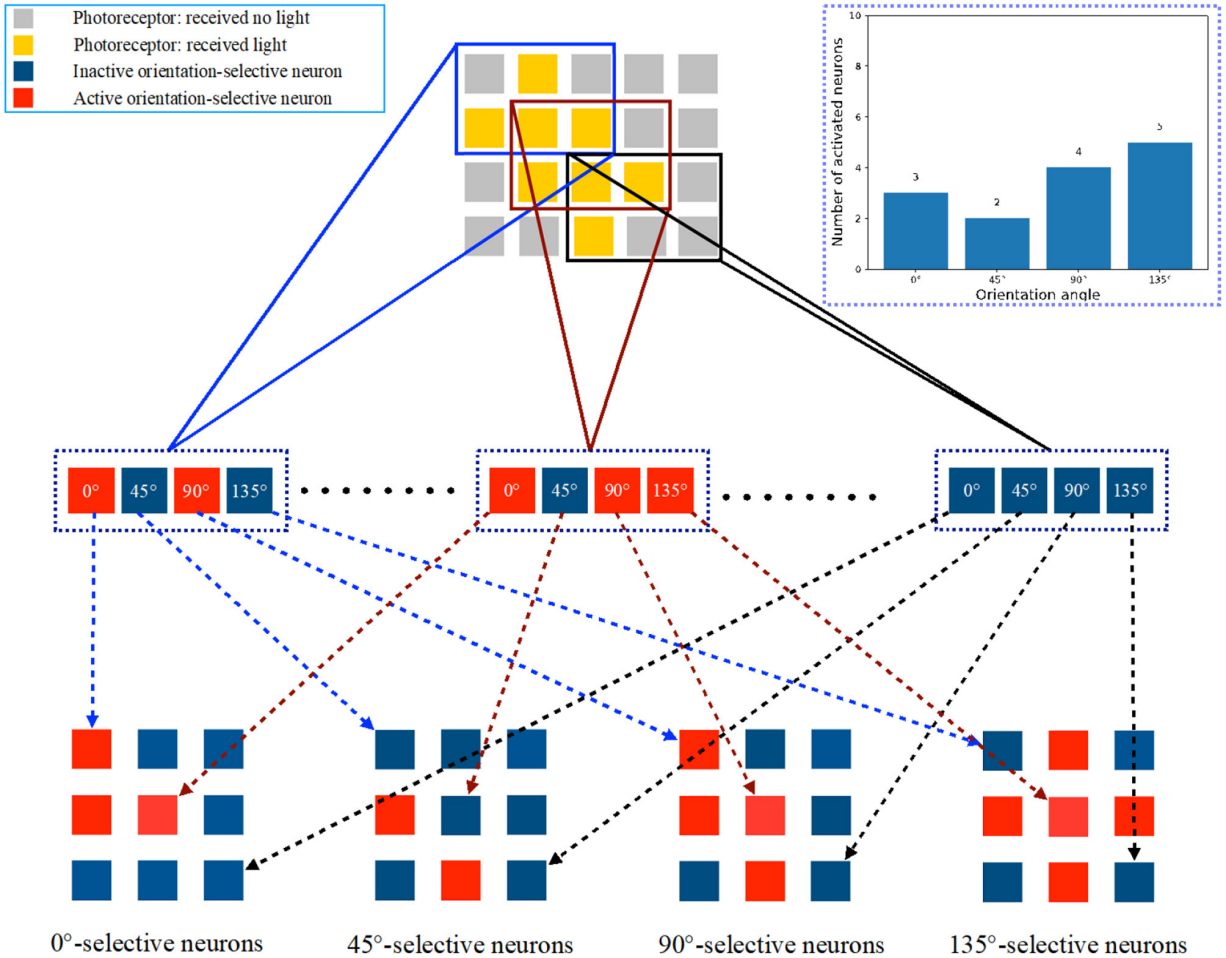
The mechanism of the single layer perceptron AVS for two-dimensional global orientation detection.

In this subsection, we describe the overall process of global orientation detection using the single-layer perceptron AVS. The system for two-dimensional global orientation detection is presented in Figure 4. Let's consider an object with a 135° orientation as an example. In Figure 4, the positions of the corresponding photoreceptors that are activated by this object are highlighted. The photoreceptors that receive light are shown in yellow, while the others are colored gray. This image, divided into 5 × 4 regions, can be further divided into 9 independent local receptive fields of size 3 × 2. Each local receptive field's photoreceptors are then connected to four different orientation-selective neurons. Therefore, there are a total of 36 orientation-selective neurons connected to the photoreceptors for this 5 × 4 image. Figure 4 illustrates three local receptive fields and their corresponding orientation-selective neurons. The three local regions are enclosed in colored frames. The activated orientation-selective neurons are depicted in red, while the inactivated ones are shown in blue. During an orientation detection process, the inputs in each local receptive field are transmitted to the four orientation-selective neurons. The local orientation information is computed independently by the four types of orientation-selective neurons.

**Figure 5:**
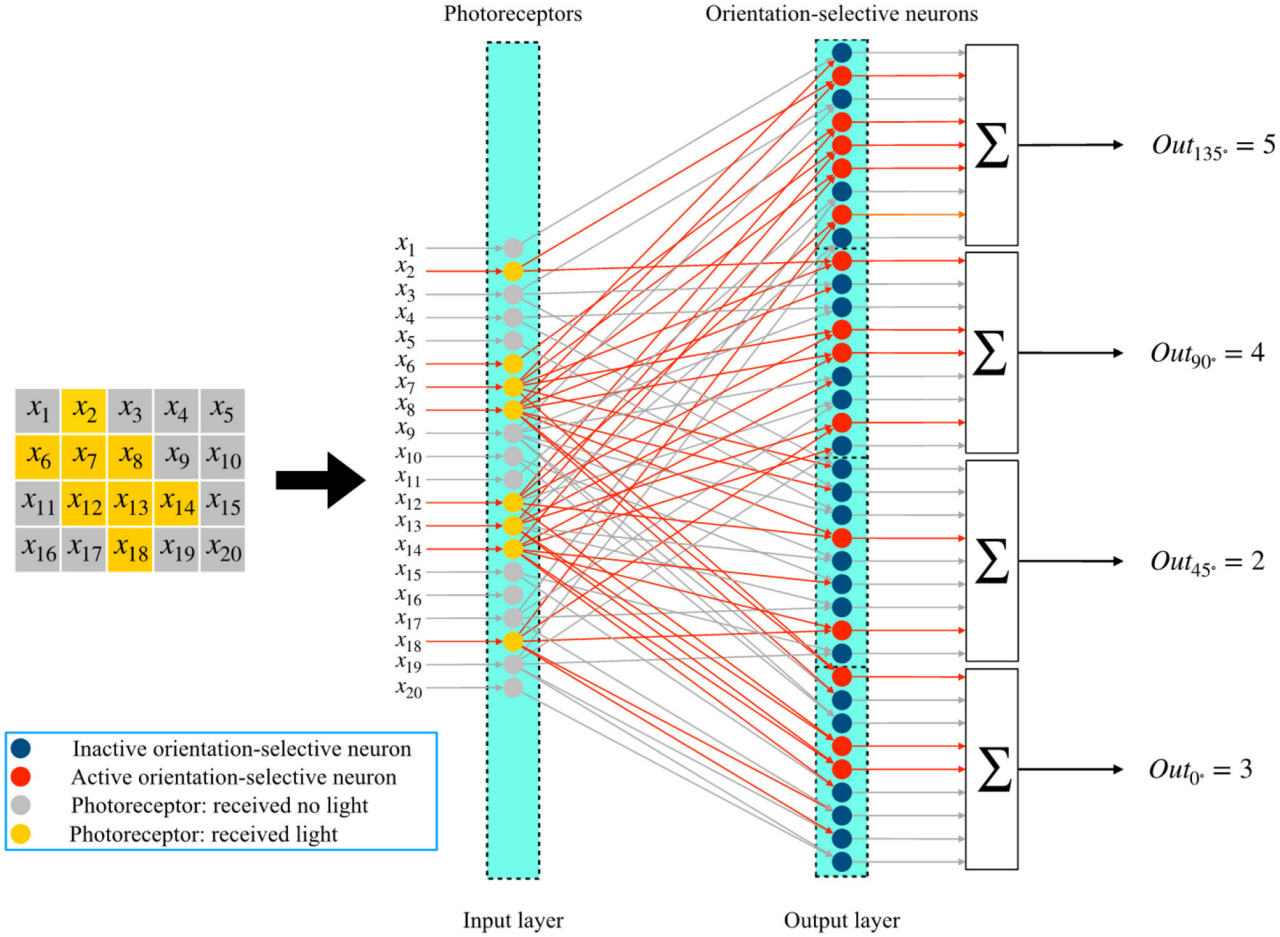
The single-layer perceptron AVS for two-dimensional global orientation detection.

The corresponding orientation-selective neurons are activated based on the effective local orientation information. For example, in the first local receptive field, only a 0°-selective neuron and a 90°-selective neuron are activated by the inputs. In the last local receptive field, no neurons are activated. By arranging the orientation-selective neurons according to their corresponding positions, the arrangements are displayed in the lower part of Figure 5. This arrangement allows us to easily determine the positions of activated neurons and the number of different types of activated neurons. The activations of the four types of orientation-selective neurons are shown in the bar chart. Since the global orientation can be determined based on the number of most activated orientation-selective neurons, the type of neurons with the highest activation count corresponds to the global orientation of the object. From the bar chart, we can observe that five 135°-selective neurons are activated five times, which is the highest activation count. Therefore, the detection result is that this object has a 135° orientation. The complete system for two-dimensional global orientation detection based on the single-layer perceptron is depicted in Figure 5. It consists of three layers: the photoreceptor layer, the local orientation-selective neuron layer, and the sum layer. The connections between the photoreceptor layer and the local orientation-selective neuron layer are not fully connected. Each local orientation-selective neuron accepts specific inputs based on the distribution characteristics of different input groups in the local receptive field. Neurons are defined as four different orientation-selective neurons. Finally, the outputs from the same type of local orientation-selective neurons are combined in a summer, where they are simply summed. This step calculates the sum of effective inputs, which corresponds to counting the number of activated neurons of that type. The four final output results represent the counts of activated neurons for the four types. In this system, effective connections and active neurons are highlighted in red. The output value of an active neuron is 1, while that of an inactive neuron is 0. The four results are consistent with the results shown in Figure 4.

## 3. Simulation results

**Figure 6:**
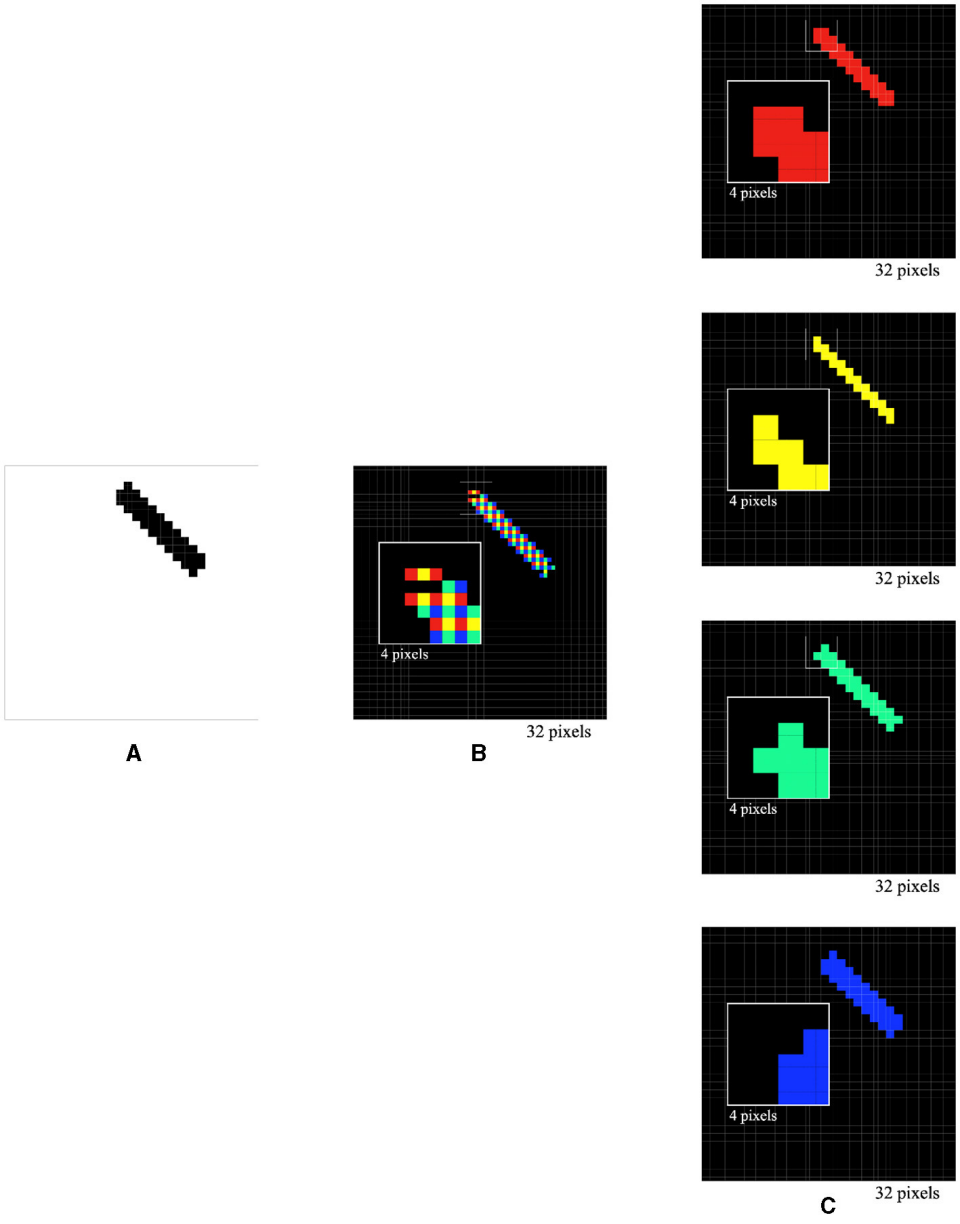
Simulated responses of the local orientation detective neurons to a line stimulus of 1 × 10 at a 135° orientation **(A)**, overall activations **(B)** and individual activations of 0°-selective neurons, 45°-selective neurons, 90°-selective neurons, and 135°-selective neurons **(C)**.

To validate the effectiveness of the single-layer perceptron AVS for global orientation detection, several computer experiments were conducted using a dataset consisting of 49,694 binary images. Each image was 1024 pixels in size (32 × 32) and contained various numbers of light spots arranged into regular objects with central or axial symmetry at specific orientation angles. For each image, we applied the four different orientation-selective neurons to each local receptive field for local orientation detection. The activations of each type of neuron were then used to infer the global orientation. To account for edge information and the 3 × 2 size of each local receptive field, the 32 × 32 images were padded with zeros on the boundary (right, left, and top), resulting in image sizes of 34 × 33. This allowed for the division of each image into 1,024 (32 × 32) local receptive fields. Consequently, a total of 4,096 (4 × 1024) orientation-selective neurons were involved in orientation detection. In the first experiment, a 1 × 10 line at a 135° orientation was placed in the images (Figure 6A). The activations of the four types of orientation-selective neurons were recorded, including the overall activations (Figure 6B) and individual activations of the 0°-selective, 45°-selective, 90°-selective, and 135°-selective neurons (Figure 6C). As shown in Figures 6B, C, only the 135° orientation-selective neuron was activated, while the others remained inactive. Therefore, the orientation-selective neuron (135°) with the highest activation count could be used to determine the global orientation of the line. By varying the lengths, angles, and positions of the line, it was consistently observed that only the 135° orientation-selective neuron was activated, regardless of these variations.

**Figure 7:**
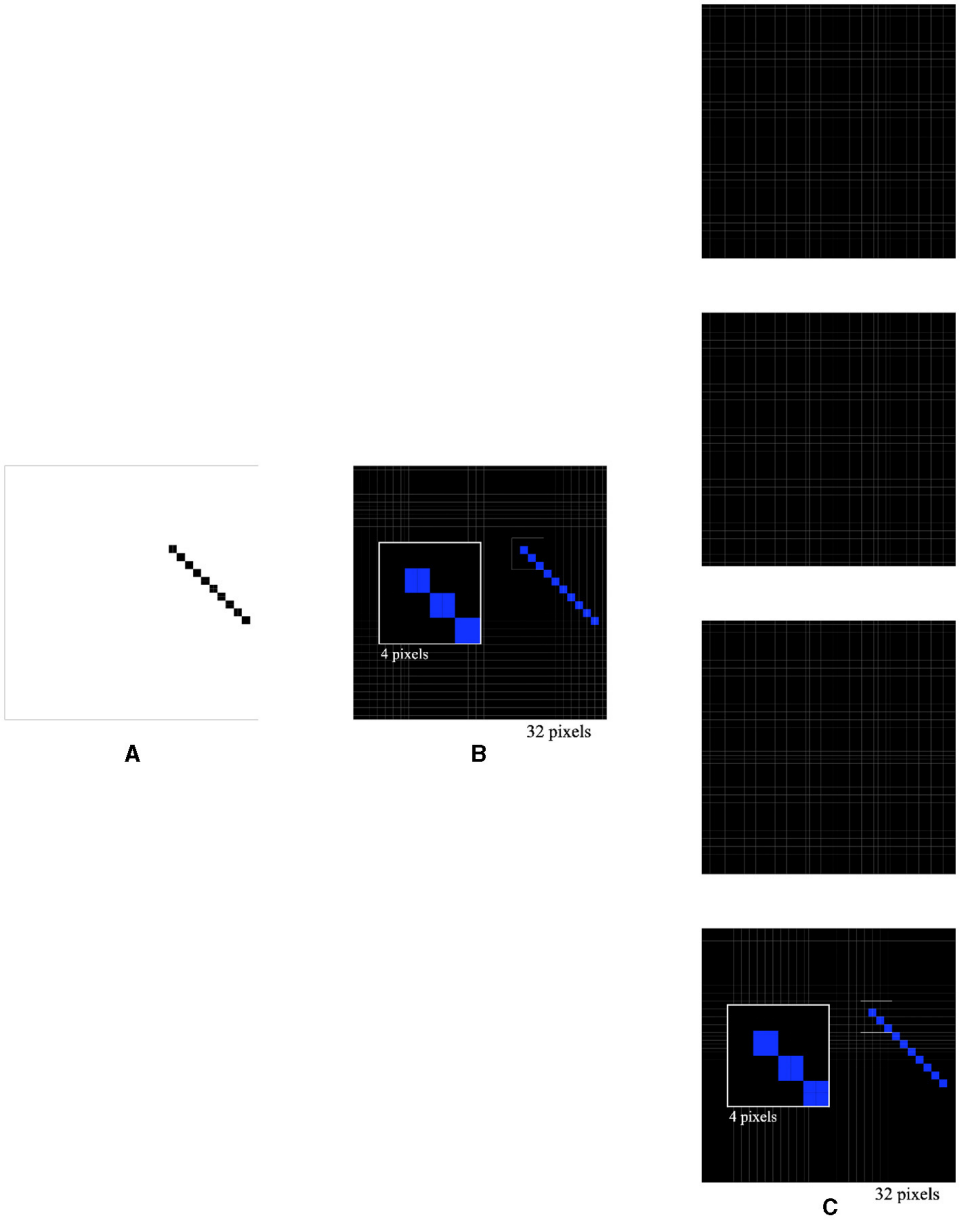
Simulated responses of the local orientation detective neurons to a bar stimulus of 4 × 10 at a 135° orientation **(A)**, overall activations **(B)**, and individual activations of 0°-selective neurons, 45°-selective neurons, 90°-selective neurons, and 135°-selective neurons **(C)**.

In the next experiment, a 135° 4 × 10 pixel bar was used as the stimulus (Figure 7A). The activations of the four types of orientation-selective neurons were recorded, including the overall activations (Figure 7B) and individual activations of the 0°-selective, 45°-selective, 90°-selective, and 135°-selective neurons (Figure 7C). Interestingly, it was observed that 28 0°-selective neurons, 20 45°-selective neurons, 29 90°-selective neurons, and 36 135°-selective neurons were activated by the 135° 4 × 10 pixel bar. This allowed for the correct determination that the bar was placed at a 135° orientation. Furthermore, by varying the lengths, widths, angles, and positions of the bar, it was consistently observed that the 135°-selective neurons had the highest activation count, indicating the correct recognition of the bar's orientation. These experiments confirmed key experimental observations from previous studies and provided explanations for those observations (Hubel and Wiesel, 1959, 1962, 1968; Kondo et al., 2016). They could prompt neuroanatomists and neurophysiologists to reexamine their findings or reconsider their experimental designs.

Additionally, the performance of the single-layer perceptron AVS for global orientation detection was evaluated using a larger image dataset, where objects of various sizes (2 to 48 pixels) were placed at different positions and angles. Each experiment was repeated 30 times, and the average results are presented in Table 1. The results demonstrate that regardless of the object's size and position, its orientation angle can be accurately recognized by the single-layer perceptron AVS.

**Table 1:** Accuracy analysis of orientation detective system.

**Object type**		**Orientation angle**			
		**0**°	**45**°	**90**°	**135**°
2 pixels	No. of samples	992	961	992	961
	Correct numbers	992	961	992	961
	Accuracy	100%	100%	100%	100%
3 pixels	No. of samples	960	960	960	960
	Correct numbers	960	960	960	960
	Accuracy	100%	100%	100%	100%
4 pixels	No. of samples	928	841	928	841
	Correct numbers	928	841	928	841
	Accuracy	100%	100%	100%	100%
8 pixels	No. of samples	1,699	2,249	1,699	2,249
	Correct numbers	1,699	2,249	1,699	2,249
	Accuracy	100%	100%	100%	100%
12 pixels	No. of samples	2,379	3,411	2,379	3,411
	Correct numbers	2,379	3,411	2,379	3,411
	Accuracy	100%	100%	100%	100%
16 pixels	No. of samples	1,319	1,489	1,319	1,489
	Correct numbers	1,319	1,489	1,319	1,489
	Accuracy	100%	100%	100%	100%
32 pixels	No. of samples	1,284	1,645	1,284	1,645
	Correct numbers	1,284	1,645	1,284	1,645
	Accuracy	100%	100%	100%	100%
≥48 pixels	No. of samples	2,515	1,275	2,515	1,275
	Correct numbers	2,515	1,275	2,515	1,275
	Accuracy	100%	100%	100%	100%

**Figure 8:**
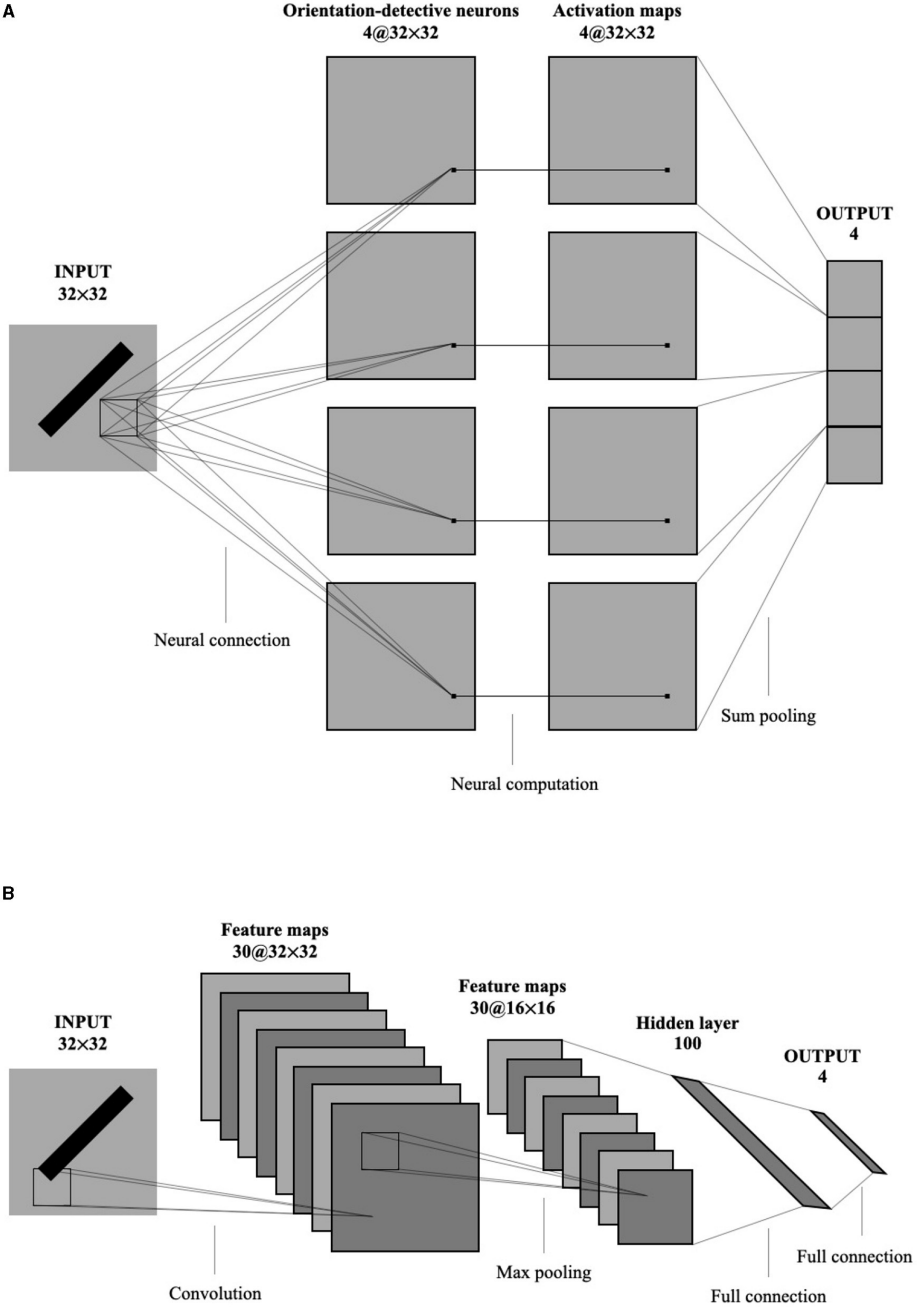
The architecture of the single-layer perceptron AVS **(A)** and CNN **(B)** used in experiments.

To compare the global orientation detection performance of the single-layer perceptron AVS with other methods, CNNs were selected due to their widespread application and success in object detection, segmentation, and recognition in images. Figure 8 illustrates the architectures of the single-layer perceptron AVS (a) and the CNN (b) used in the experiments. The CNN used in the experiments follows a typical architecture for handwritten character recognition (Saito, 2018). It consists of 7 layers:

1. Convolutional layer: It employs 30 filters of size 3 × 3 to generate 30 feature maps of size 32 × 32.
2. Pooling layer: It performs 2 × 2 maximum pooling on the feature maps.
3. Affin layer: This layer includes a fully connected network that maps the inputs from the previous layer (8,192 inputs from the 30 × 16 × 16 feature maps) to a hidden layer of size 100, and then to an output layer of size.
4. The activation function of nodes in Affin layer is standard sigmoid function. The learning was performed on both convolution layer and Affin layer.

Since the input images are 32 × 32 pixels, the CNN has a total of 1024 inputs. The convolutional layer produces 30 feature maps of size 32 × 32. After applying 2 × 2 maximum pooling, the inputs to the fully connected network are reduced to 8,192 (30 × 16 × 16). The fully connected network then maps these inputs to the hidden layer of size 100 and finally to the output layer of size 4.

On the other hand, the single-layer perceptron AVS has only two layers:

1. Perceptron layer: It consists of four types of orientation-selective neurons, with a total of 4096 (4 × 32 × 32) local orientation detection neurons. This layer generates four sets of 32 × 32 local orientation feature maps.
2. Summing pooling layer: This layer sums the four sets of local orientation feature maps to produce four output values.

In comparison to the CNN with 820,004 parameters, the single-layer perceptron AVS only has 12 parameters (4 × 3) for the local orientation detection neurons. This significant reduction in parameters results in substantial savings in computational cost.

**Figure 9:**
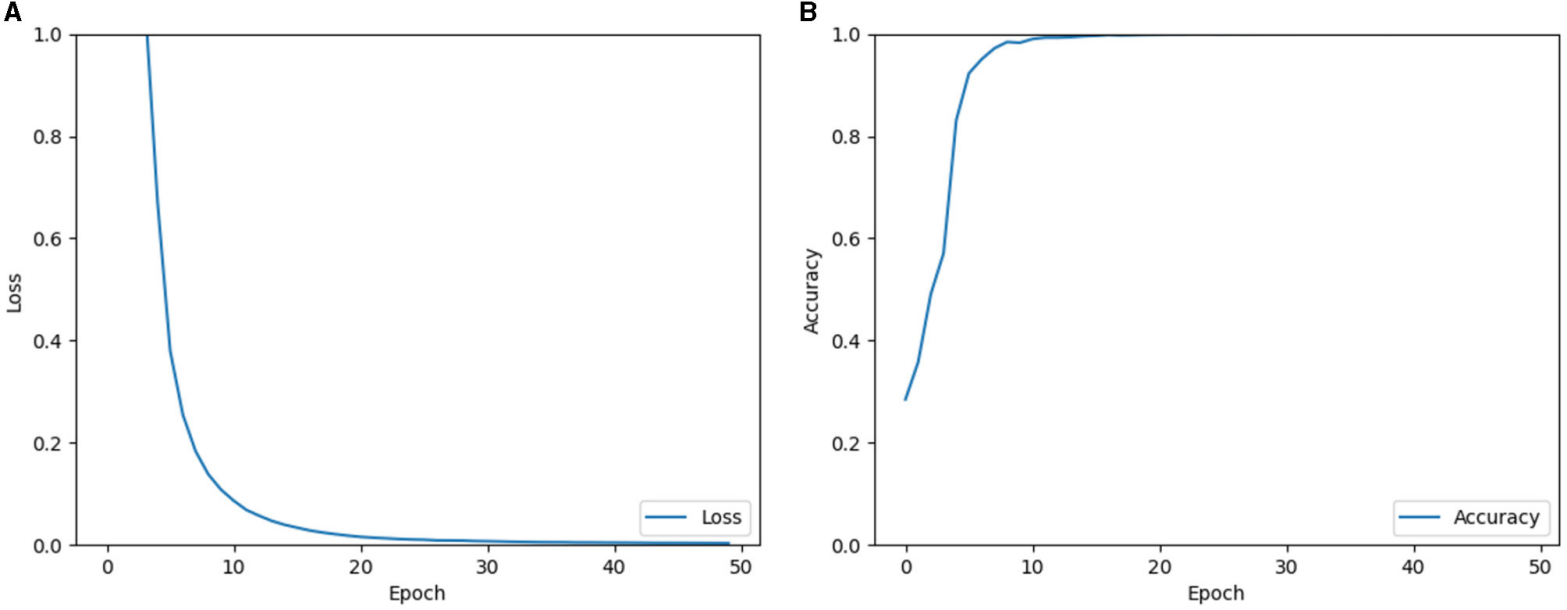
Learning results of loss **(A)** and accuracy **(B)** of the CNN.

Therefore, the single-layer perceptron AVS offers a simpler architecture with a smaller number of parameters, making it computationally efficient compared to the CNN. In the experiments, the CNN was trained for global orientation detection using a dataset of 15,000 samples, while 5,000 samples were used for testing. The objects in the dataset varied in size from 2 pixels to 256 pixels, had different shapes, and were randomly placed. The CNN was trained using back-propagation with the Adam optimizer. Figure 9 displays the learning results of the CNN, showing the loss and accuracy during the training process. From the learning curves, it can be observed that the CNN successfully learned the task of orientation detection, achieving a high identification accuracy of 99.997%. This performance indicates that the CNN performed also well in comparison to the single-layer perceptron AVS, which achieved 100% accuracy without the need for training.

The single-layer perceptron AVS possesses several advantages over CNN in various aspects:

Parameter efficiency: The single-layer perceptron AVS requires fewer parameters compared to CNN. While CNNs become deeper with millions of parameters that need to be calculated and optimized, the single-layer perceptron AVS remains compact.

Prior knowledge utilization: The single-layer perceptron AVS can leverage prior knowledge about the system and task, allowing for learning from good initial values. In contrast, CNNs typically start from random initial values and lack the ability to incorporate prior knowledge directly.

Convergence guarantee: The perceptron is specifically designed to solve linearly separable binary classification problems. The perceptron algorithm iteratively updates its weights to find a linear decision boundary (hyperplane) that can classify linearly separable binary classification problems correctly. The fact that the local orientation detection problem can be solved by the perceptron (Figure 2) means that the local orientation detection problems are linearly separable and the data points representing different local orientations can be separated by a hyperplane in the feature space. Therefore, the single-layer perceptron AVS for orientation detection is guaranteed to converge within an upper bound on the number of iterations. CNNs, on the other hand, often require significant learning time and are prone to getting stuck in local minima as it is shown in Table 2.

**Table 2:** Comparison between CNN and the single-layer perceptron AVS.

	**Layer**	**Parameters**	**Learning cost**	**Reasoning**	**Bio-soundness**	**Noise resistance**
CNN	≥7	820.004	High	Black Box	Low	Low
AVS	2	12	No	Reasonable	High	High

Interpretability and explainability: The learning process of the single-layer perceptron AVS is more transparent and understandable compared to CNN. The results and predictions of the single-layer perceptron AVS are traceable and explainable, whereas CNN learning is often considered a black box with non-transparent results.

Simple hardware implementation: The hardware implementation of the single-layer perceptron AVS is simpler and more efficient compared to CNN, as it requires only two layers instead of the hundreds of layers typically found in CNNs. The single-layer perceptron AVS stays true to the concept of locally-sensitive, orientation-selective neurons, while CNNs often overlook this essential concept.

Biological plausibility: The single-layer perceptron AVS aligns closely with the visual system concepts proposed by Hubel and Wiesel, making it more biologically sound for orientation detection and other visual tasks. In contrast, CNNs, despite their similarities in connecting units to local receptive fields, do not fully incorporate the concept of orientation-selective neurons and cannot be regarded as true “neural” networks.

Adding noise to test the noise resistance of CNN and the single-layer perceptron AVS is a common practice in evaluating the robustness of these systems to noisy inputs. Noise resistance is an important characteristic for any image processing or pattern recognition system as it determines how well the system can handle inputs with random variations. In our experiments, for each image in the testing dataset, noise is generated by randomly selecting a certain percentage of pixels. The percentage of pixels selected ranges from 5 to 30%. For each selected pixel, if it was originally 0, it is changed to 1, and if it was originally 1, it is changed to 0. The images with noise were fed to both systems, and their noise resistance was compared.

Table 3 summarizes the noise resistance results. It can be seen that when subjected to a 5% noise level, CNN's identification accuracy dropped to 90%, while the single-layer perceptron AVS dropped to 96%. As the noise level increased to 30%, CNN's identification accuracy dramatically decreased to 35%, whereas the single-layer perceptron system maintained a 43% identification accuracy, demonstrating superior noise resistance. In summary, the single-layer perceptron AVS exhibits advantages in parameter efficiency, utilization of prior knowledge, convergence guarantee, interpretability, and hardware implementation efficiency compared to CNN. It also shows higher noise resistance in the presence of noisy inputs.

**Table 3:** Accuracy of CNN and AVS.

**Noise**	**0%**	**5%**	**10%**	**15%**	**20%**	**25%**	**30%**
CNN	99.887%	90.783%	74.441%	59.108%	47.547%	39.866%	35.343%
AVS	100%	96.571%	85.562%	71.490%	59.716%	49.924%	43.452%

## 4. Conclusion and discussion

This paper proposed a novel orientation detection mechanism-based single-layer perceptron AVS. By introducing the concept of local receptive fields and implementing local orientation detective neurons with a single-layer perceptron, the system achieved global orientation detection by determining the most activated orientation detective neuron. The effectiveness of the system was demonstrated through extensive computer experiments, showing excellent recognition accuracy regardless of object size, location, and orientation. The mechanism and the mechanism-based AVS exhibit desirable properties that can be applied to various artificial visual perception systems and are reminiscent of the human visual system. They can serve as a framework for understanding fundamental phenomena in visual perception, such as direction perception, movement direction perception, movement speed perception, and binocular vision perception. Additionally, they provide a functional framework for visual computing in the primary visual cortex, shedding light on how visual input is processed and organized across different stages of the visual system.

Furthermore, the mechanism and the mechanism-based AVS offer insights into encoding sensory information in cortical circuits, which can extend to other sensor systems like smell, taste, and touch. Although the mechanism and AVS are based on simplified models and overlook certain detailed functions of the visual system and the brain, they provide a quantitative explanation for many known neurobiological visual phenomena and experiments. They may also prompt neuroanatomists and neurophysiologists to reevaluate their observations or conduct new experiments to uncover corresponding structures and functions. Conversely, advancements in biological sciences can contribute to further modifications of the mechanism and the mechanism-based AVS. The paper also compared the performance of the single-layer perceptron AVS with traditional CNNs for orientation detection tasks, demonstrating the superiority of the single-layer perceptron AVS in terms of recognition accuracy, noise immunity, computation and learning costs, hardware implementation, reasoning, bio-soundness, and other aspects. Overall, the proposed mechanism and the mechanism-based AVS offer a promising approach to orientation detection and lay the foundation for future research and advancements in the field of visual perception. But, we must point out that the proposed system lacks generality, which means it is limited in its application to only visual perceptions, such as orientation perception, movement direction perception, movement speed perception, binocular vision perception and other sensor systems like smell, taste, and touch.

## Data availability statement

The raw data supporting the conclusions of this article will be made available by the authors, without undue reservation.

## Author contributions

HT, TC, and JY designed the study, formulated research questions, drafted the manuscript, revised it based on feedback, and prepared the final version for submission. BL collected the data, conducted experiments, and performed the analyses. YT and ZT conceptualized the study and supervised the project. All authors contributed to the article and approved the submitted version.

## References

[B1] Gazzaniga M. S. (2000). Cognitive Neuroscience: A Reader. Blackwell Publishing.

[B2] Hubel D. H. (1982). Exploration of the primary visual cortex, 1955–78. Nature 299, 515–524. 6750409 10.1038/299515a0

[B3] Hubel D. H. Wiesel T. N. (1959). Receptive fields of single neurones in the cat's striate cortex. J. Physiol. 148, 574. 14403679 10.1113/jphysiol.1959.sp006308 PMC1363130

[B4] Hubel D. H. Wiesel T. N. (1962). Receptive fields, binocular interaction and functional architecture in the cat's visual cortex. J. Physiol. 160, 106. 14449617 10.1113/jphysiol.1962.sp006837 PMC1359523

[B5] Hubel D. H. Wiesel T. N. (1968). Receptive fields and functional architecture of monkey striate cortex. J. Physiol. 195, 215–243. 4966457 10.1113/jphysiol.1968.sp008455 PMC1557912

[B6] Kandel E. Schwartz J. Jessell T. (1991). Principles of Neural Science. East Norwalk, CT: Appleton and Lange.

[B7] Kondo S. Yoshida T. Ohki K. (2016). Mixed functional microarchitectures for orientation selectivity in the mouse primary visual cortex. Nat. Commun. 7, 1–16. 10.1038/ncomms13210 27767032 PMC5078743

[B8] Lee Y. Jung K. Lee H. (2020). “Human response characteristics according to the location of visual stimuli,” in International Conference on Applied Human Factors and Ergonomics (Springer), 322–327.

[B9] Li B. Todo Y. Tang Z. (2021). “The mechanism of orientation detection based on local orientation-selective neuron,” in 2021 6th International Conference on Computational Intelligence and Applications (ICCIA) (IEEE), 195–199.

[B10] McCulloch W. S. Pitts W. (1943). A logical calculus of the ideas immanent in nervous activity. Bull. Math. Biophys. 5, 115–133. 2185863

[B11] Medina J. J. Hanlon D. (2009). 12 Principles for Surviving and Thriving at Work, Home and School. Pear Press.

[B12] Rosenblatt F. (1958). The perceptron: a probabilistic model for information storage and organization in the brain. Psychol. Rev. 65, 386. 13602029 10.1037/h0042519

[B13] Saito K. (2018). Deep Learning from Scratch-Natural Language Processing. Tokyo: O'Reilly Japan, Inc.

[B14] Todo Y. Tang Z. Todo H. Ji J. Yamashita K. (2019). Neurons with multiplicative interactions of nonlinear synapses. Int. J. Neural Syst. 29, 1950012. 10.1142/S0129065719500126 31189391

[B15] Vanston J. E. Strother L. (2017). Sex differences in the human visual system. J. Neurosci. Res. 95, 617–625. 10.1002/jnr.23895 27870438

[B16] Veeser S. Cumming D. (2017). Object Position and Orientation Detection System. US Patent 9536163. United States: Patent Application Publication.

